# Supplementary material for: A natural history museum visitor survey of perception, attitude and knowledge (PAK) of microbes and antibiotics
Source: PLoS One. 2021 Sep 22;16(9):e0257085. doi: 10.1371/journal.pone.0257085 (PMC8457478; doi:10.1371/journal.pone.0257085)
Supplement: S1 File — The literature on public knowledge of antibiotics is substantial. The comparisons span a large number of countries and demgraphies. (DOCX) [file pone.0257085.s001.docx]

**Supplemental File 1.**

The literature on public knowledge of antibiotics is substantial. Our canvassing of the literature revealed nearly a hundred surveys attempting to address PAK in about 60 different countries (see **Supplemental Table 1.1**). Several questions on these surveys are consistent from survey to survey. For instance, the following question (or a version of it) appeared in almost all surveys - “Answer TRUE/FALSE: Antibiotics are effective in killing viruses”. We compiled some of the results from these surveys (**Supplemental Table 1.1**). The first compilation shows the percentage, method of survey and country where the study queried about the effectiveness of antibiotics on flu, viruses or the common cold. The data shown in **Supplemental Table 1.1** and **Supplemental Figure 1.1** show the literature survey for this category of question.

**Country N Q/W ref % wrong and comment**

Australia 155 Q 53 35% not confident when not to prescribe antibiotics

Australia 229 Q 6 56% think antibiotics can treat viruses

Bahrain 310 Q 3 69% think AB can treat viruses

Bangladesh 350 Q 56 33% think AB can treat viruses

Bangladesh 600 Q 16 66% discontinue AB when they feel better

Bangladesh 760 Q 68 53.46% stopped taking AB as soon as symptoms subside

Bangladesh 250 Q 41 58% think AB can treat viruses

Bangladesh 250 Q 41 57% think AB can treat colds

Barbados 507 Q 53 47% answer that cold and flu can be treated with antibiotics

Bhutan 692 Q 43 65% score unsatisfactory on four antibiotic questions

Cameroon 402 Q 14 88.3% think ABs can be used on all infections including viral

China 1002 W 53 61% answer that cold and flu can be treated with antibiotics

China 1086 W 50 44% identify viruses as being susceptible to antibiotics

China 2390 Q 57 92% believed that antibiotics can control viruses

China (MS) 2500 Q 17 44% cannot state properly that viruses cannot be treated with AB

Egypt 511 Q 53 76% answer that cold and flu can be treated with antibiotics

Ethiopia 384 Q 58 83% antibiotics speed up the recovery from coughs and colds

France 200 Q 66 24% believed that antibiotics can control viruses

Georgia 250 Q 22 55 % also agreed that antibiotics are effective against viruses.

Germany 977 Q 60 31% suggest AB can kill viruses

Germany 2000 Q 59 31% suggest AB can kill viruses

Greece 7704 Q 31 40% of respondents thought ABs worked on viruses

Hong Kong 1255 Q 36 54.0% identified cold and flu as treatable with antibiotics.

India 1023 W 53 75% answer that cold and flu can be treated with antibiotics

India 145 Q 61 78% answer that cold and flu can be treated with antibiotics

India 491 Q 12 80% answer that cold and flu can be treated with antibiotics

India 55 W 13 68% answer that cold and flu can be treated with antibiotics

India (MS) 382 Q 19 64% think viruses can be treated with antibiotics

Indonesia 1027 W 53 63% answer that cold and flu can be treated with antibiotics

Indonesia 559 Q 72 70% believed that antibiotics are effective against viruses

Iran 500 Q 1 45% believed that antibiotics are effective against viruses,

Iraq 680 Q 62 30% believed that antibiotics are effective against viruses,

Italy 1247 Q 7 33% declared that antibiotics are useful for viral infections,

Italy 1247 Q 7 21% declared that antibiotics cure inflammation

Italy 1247 Q 7 14% stop treatment with antibiotic prematurely

Italy 797 Q 63 40% believed that antibiotics useful for fever, cold, and flu

Italy 913 W 51 22% did not know when it was appropriate to use antibiotics

Italy (MS) 1050 Q 38 20% Medical Students say ABs work on viruses

Japan 3390 Q 21 25% did not know that antibiotics cannot kill viruses

Jordan 1141 Q 39 67% believed that antibiotics treat common cold and cough

Kosovo 811 Q 49 43% think that antibiotics are effective against viral infections

Kuwait 770 Q 67 47% low knowledge regarding resistance of antibiotics

Lebanon 400 Q 18 51% believed that antibiotics are effective against viruses,

Lebanon 500 Q 28 74% did not know that antibiotics are not anti-viral

Lithuania 1005 Q 69 47% thought AB could treat viral infections

Lithuania 1005 Q 33 50% answer that cold and flu can be treated with antibiotics

Macedonia 500 Q 2 60% did not know that antibiotics cannot cure viral infections

Malaysia 250 Q 40 83% think viruses can be treated with AB

Malaysia 383` Q 25 75% of respondents thought ABs worked on viruses

Malaysia 508 Q 25 67% thought that antibiotics are also used to treat viral infections

Malaysia 421 Q 10 76% antibiotics are helpful in treating cold, cough and fever

Mexico 1001 W 53 61% answer that cold and flu can be treated with antibiotics

Mongolia 540 Q 42 83% antibiotics should be employed for fever, cold, and flu

Namibia 446 Q 34 64% thought that antibiotics were effective against viruses

Netherlands 800 W 8 48% Antibiotics are effective in treating infections caused by viruses

Nigeria 430 Q 64 66 % expectation to be prescribed an antibiotic for cold

Nigeria 664 Q 53 44% answer that cold and flu can be treated with antibiotics

Norway 877 Q 65 27% think that antibiotics are effective against viral infections

Pakistan 400 Q 70 57% stated that antibiotics could cure all types of infections

Palestine 385 Q 37 69% think flu can be treated with AB

Pan-Islamic 1401 Q 6 63% believed that antibiotics cure common cold and flu

Poland 5004 Q 26 60% of respondents believed antibiotics kill viruses

Poland 5004 Q 26 60%of respondents believed antibiotics kill viruses

Poland 891 Q 29 44% of respondents thought ABs worked on viruses

Portugal 349 Q 54 21% recognize AB as useful against virus

Romania 996 Q 45 40% claim AB are for viral infections

Russia 1007 W 53 67% answer that cold and flu can be treated with antibiotics

Samoa 112 Q 30 81% antibiotics as a useful treatment for cold and flu

Saudi Arabia 1966 Q 48 24% believed antibiotics work on viruses

Saudi Arabia 400 Q 47 63% poor knowledge of AB

Saudi Arabia 1310 Q 52 71 % reported that they did not finish the antibiotic course

SE Asia 2141 Q 15 88% confused anti-inflammatory compounds with antibiotics

Serbia 510 Q 53 68% answer that cold and flu can be treated with antibiotics

South Africa 1002 W 53 69% answer that cold and flu can be treated with antibiotics

South Africa 386 Q 55 55% incorrectly thought that antibiotics are also used to treat viral

South Korea 1117 Q 23 70% did not know that antibiotics are ineffective in treating colds

Sudan 518 Q 53 80% answer that cold and flu can be treated with antibiotics

Sweden 1000 Q 5 27% of respondents think antibiotics can kill viruses

Switzerland 1260 W 44 56% of respondents think antibiotics work on flu and common cold

Tanzania 290 Q 71 65% think AB can treat viruses

Thailand. >10000 Q 11 81% of respondents thought ABs worked on viruses

Trinidad 699 Q 32 35% think viruses can be treated with antibiotics

Turkey 100 Q 76 36% think AB can kill viruses

United Kingdom 625 W 74 45% thought AB would be effective on viruses

United Kingdom 2283 Q 73 35% thought that antibiotics kill viruses/treat viral infections

USA 1004 Q 35 27% state AB can kill viruses

USA 1004 Q 35 36% suggest that AB can kill viruses

USA 1004 Q 35 37% state AB can kill viruses

USA 215 W 9 40% suggest that AB can kill viruses

USA 25 Q 4 56% think AB can treat flu

USA 10,780 Q 75 32% taking antibiotics during a cold prevented more serious illness

Viet Nam 1000 W 53 62% answer that cold and flu can be treated with antibiotics

**Supplemental Table 1.1. Percentage of answers indicating misunderstanding of antibiotics.** The country surveyed and sample size (N) are shown in the first and second columns. Whether the survey was in person questionnaire (Q) or web-based (W) is indicated in the third column. The reference number (see literature list below) for the study is shown in the fourth column. The percentage of answers indicating a misunderstanding of antibiotics and a comment on the nature of the misunderstanding is given in the last column.

When “viruses” is inserted into the question the percentage of answers indicating a misunderstanding of antibiotics ranges from 20% to 90%. When “flu” is inserted into the question the range of percentage of wrong answers is 30% to 93%. Interestingly there is a general trend of lower percentage of answers indicating a misunderstanding of antibiotics from developed countries versus developing countries (**Supplemental Figure 2.1**). Our kiosk survey results are indirectly comparable to the antibiotics/viruses/flu question discussed above and is discussed in detail in the text.


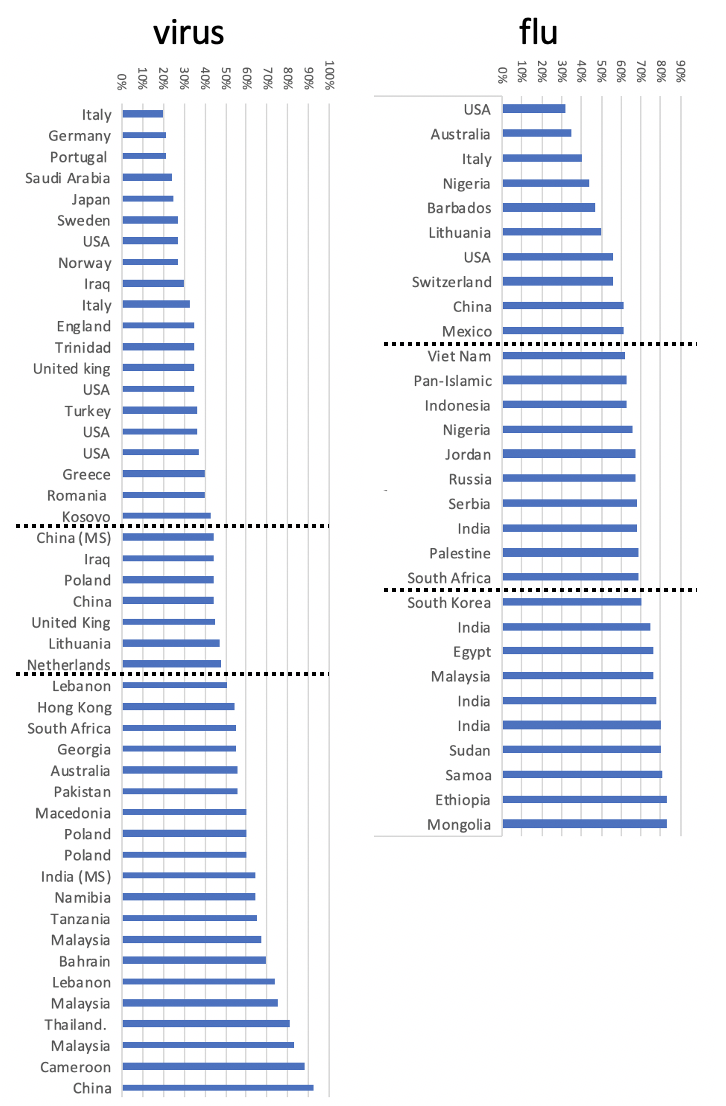


**Supplemental Figure 1.1. Histograms showing the percentage of wrong answers for utility of antibiotics.** In general the question asked is TRUE/FALSE – Antibiotics are effective at treating viruses or TRUE/FALSE – Antibiotics are effective at curing flu and colds. The summary for the flu question is on the right under “flu” and the summary for the viruses question is on the left under “viruses”. The dotted lines are arbitrarily drawn lines to show the top and bottom twenty countries for viruses and top and bottom ten countries for flu.

Another set of questions that was relatively consistent across surveys in the literature involved correctly labeling a medicinal compound (**Supplemental Table 1.2**) as an antibiotic such as “Answer TRUE/FALSE penicillin is an antibiotic” or Answer TRUE/FALSE aspirin is an antibiotic.”

**Country N aspirin paracet Actual AB reference #**

Bhutan 692 21 32 43 93

India (MS) 382 37 35 90 81

Italy 1247 46 94 78

Lebanon 500 12.7 9.1 53.1 84

Malaysia 250 17 39 91

Malaysia 383 33.4 62 82

Mexico 101 45 44 89 79

Nepal 220 15 29 85

NZ India 130 67 86

NZ Egypt 102 28 83 86

NZ S. Korea 104 41 37 86

Nigeria 430 53 29 85 77

Saudi (MS1) 130 6 8 89 92

Saudi (MS2) 60 30 36 34 92

Saudi (MS) 347 29.7 16.7 26.8 94

South Africa 386 8 64 89

Tanzania 292 29 28 58 83

Trinidad 753 9 10 83 87

Turkey 100 4 22 63 76

Germany 977 10 10 86 90

Spain (MS) 578 7 85 88

UK (MS) 583 2 96 80

**Supplemental Table 1.2. Representative surveys from the literature addressing antibiotic recognition.** The country is given in the “Country” column. (MS) indicates medical students. The New Zealand survey included people from India, South Korea and Egypt. The sample size is given in the N column. The values under “aspirin” and “paracet” (Paracetemol) are frequencies of wrong answers (ie that aspirin or Paracetemol are antibiotics). The values under Actual AB are the frequencies of correct answers. The antibiotic most prominently used as an example was penicillin. The source of the data is in the “Reference #” column.

Results of the literature survey for answers to the second category of questions on recognizing antibiotics is shown in **Supplemental Table 1.2** and **Supplemental Figure 1.2**. These results are directly comparable to the results from the two kiosk surveys because the questions on surveys in the literature overlap with the question about name recognition of antibiotics in the literature.

When penicillin is used in the question, the percent of correct answers in the literature survey ranges from 28% to 95%. When aspirin is the query word, the percent of correct answers in the literature survey ranges from 47% to 96%. These ranges can be compared to the ranges we infer from Survey 1 (Penicillin: 70% to 83%; average 78%+/- 4.2%; Aspirin: 24% to 46%; average 29% +/- 7.4%) and from Survey 2 (Penicillin: 70% to 83%; average 78%+/- 4.2%; Aspirin: 24% to 46%; average 29% +/- 7.4%). While the ranges in the literature are large there is general overlap with a global average of 54% wrong answers. The general conclusion that there is poor understanding globally about antibiotics is sustained by the literature search.


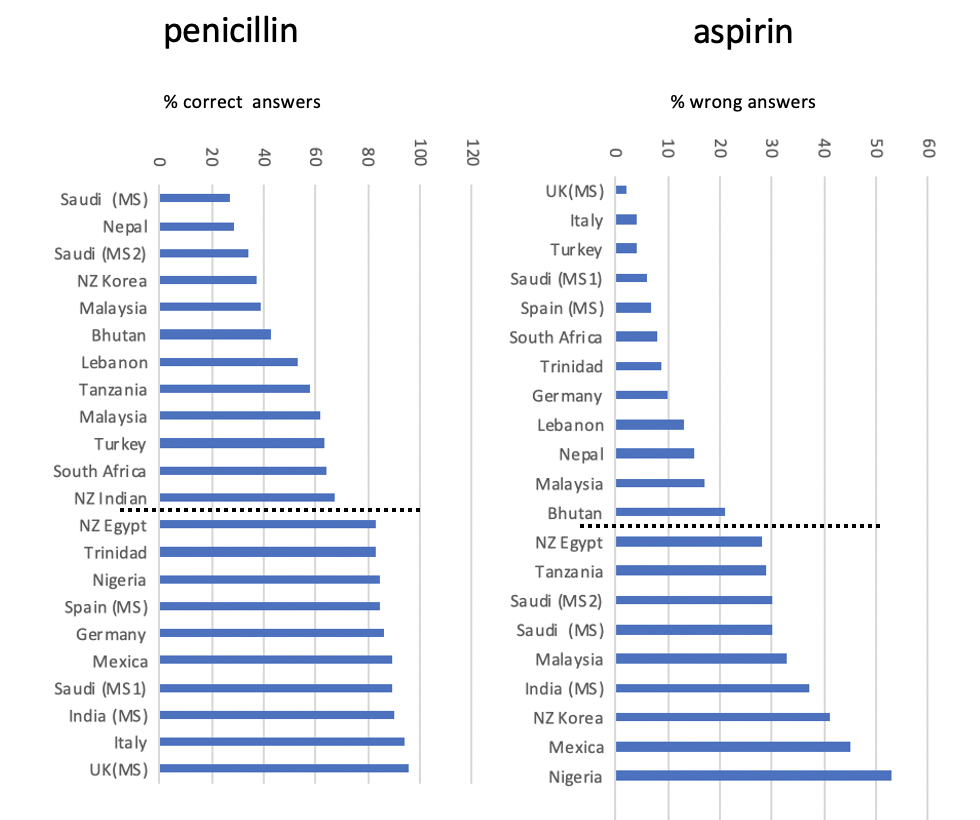


**Supplemental Figure 1.2. Histograms showing survey answers for recognition of penicillin and aspirin as antibiotics.** For the left graph, when respondents were asked “Is penicillin an antibiotic?”, the percentages shown here are correct answers. For the right graph, when respondents were asked “Is aspirin an antibiotic?”, the percentages shown here are incorrect answers. Abbreviations – MS=medical students; NZ=New Zealand (for three of the values in the figure respondents from Egypt, India and Korea were surveyed in New Zealand).

**References**

1. Al-Yasseri, Berq J. Hadi, and Nibras A. Hussain. "Public Knowledge and Attitudes Towards Antibiotics Use and Resistance in Baghdad, Iraq: A Survey Conducted in Outpatient Department of University Teaching Hospital." The Open Public Health Journal 12, no. 1 (2019).

2. Alili-Idrizi, Edita, Merita Dauti, and Ledjan Malaj. "Validation of the parental knowledge and attitude towards antibiotic usage and resistance among children in Tetovo, the Republic of Macedonia." Pharmacy practice 12, no. 4 (2014).

3. Alqallaf, Sayed Mahmood, and Win Winit‑Watjana. "Patients’ awareness of antibiotic use in Bahraini health centers and community pharmacies." Archives of Pharmacy Practice Vol 6, no. 2 (2015).

4. American views on AB resistance. Zogby poll; Research!America (USA), 2018.

5. André, Malin, Åsa Vernby, Johanna Berg, and Cecilia Stålsby Lundborg. "A survey of public knowledge and awareness related to antibiotic use and resistance in Sweden." Journal of Antimicrobial chemotherapy 65, no. 6 (2010): 1292-1296.

6. Azeem, Mohammad, Mohamed Tashani, Osamah Barasheed, Leon Heron, Grant A Hill-Cawthorne, Elizabeth Haworth, Dominic E Dwyer, Harunor Rashid, and Robert Booy. "Knowledge, attitude and practice (KAP) survey concerning antimicrobial use among Australian Hajj pilgrims." Infectious Disorders-Drug Targets (Formerly Current Drug Targets-Infectious Disorders) 14, no. 2 (2014): 125-132.

7. Bert, Fabrizio, Maria R. Gualano, Renata Gili, Giacomo Scaioli, Emanuela Lovato, Italo F. Angelillo, Silvio Brusaferro et al. "Knowledge and attitudes towards the use of antibiotics in the paediatric age group: a multicenter survey in Italy." The European Journal of Public Health 27, no. 3 (2017): 506-512.

8. Cals, Jochen WL, Dennis Boumans, Robert JM Lardinois, Ralph Gonzales, Rogier M. Hopstaken, Christopher C. Butler, and Geert-Jan Dinant. "Public beliefs on antibiotics and respiratory tract infections: an internet-based questionnaire study." Br J Gen Pract 57, no. 545 (2007): 942-947.

9. Carter, Rebecca R., Jiayang Sun, and Robin LP Jump. "A survey and analysis of the American public's perceptions and knowledge about antibiotic resistance." In Open forum infectious diseases, vol. 3, no. 3. Oxford University Press, 2016.

10. Chan, Giin Cherng, and Swee Fong Tang. "Parental knowledge, attitudes and antibiotic use for acute upper respiratory tract infection in children attending a primary healthcare clinic in Malaysia." Malaysian Family Physician 2, no. 1 (2012): 5.

11. Chanvatik, Sunicha, Hathairat Kosiyaporn, Angkana Lekagul, Wanwisa Kaewkhankhaeng, Vuthiphan Vongmongkol, Apichart Thunyahan, and Viroj Tangcharoensathien. "Knowledge and use of antibiotics in Thailand: A 2017 national household survey." PloS one 14, no. 8 (2019).

12. Chinnasami B, Sadasivam K, Ramraj B, Pasupathy S. Knowledge, attitude and practice of parents towards antibiotic usage and its resistance. Int J Contemp Pediatr 2016; 3(1): 256-61.

13. Dharman, Dhanya, Deepa Manohar, Shaiju S. Daran, Ashima Basher, and Ashita Ajith. "Knowledge and attitudes towards appropriate antibiotics usage among students-a survey." International Journal of Research in Hospital and Clinical Pharmacy 1, no. 3 (2019): 88-90.

14. Ekambi, Grace-Ange Elong, Cecile Okalla Ebongue, Ida Calixte Penda, Emmanuel Nnanga Nga, Emmanuel Mpondo Mpondo, and Carole Else Eboumbou Moukoko. "Knowledge, practices and attitudes on antibiotics use in Cameroon: Self-medication and prescription survey among children, adolescents and adults in private pharmacies." PloS one 14, no. 2 (2019).

15. Haenssgen, Marco J., Nutcha Charoenboon, Giacomo Zanello, Mayfong Mayxay, Felix Reed-Tsochas, Yoel Lubell, Heiman Wertheim et al. "Antibiotic knowledge, attitudes and practices: new insights from cross-sectional rural health behaviour surveys in low-income and middle-income South-East Asia." BMJ open 9, no. 8 (2019): e028224.

16. Hasan, SM Raquibul, Md Mokarram Hossain, Raushanara Akter, SM Hasibul Karim, Shamsul Haque, Md Kamaluddin, and Abdul Ghani. "Pattern of antibiotics use at the primary health care level of Bangladesh: survey report-1." Stamford Journal of Pharmaceutical Sciences 2, no. 1 (2009): 1-7.

17. Huang, Ying, Jiarui Gu, Mingyu Zhang, Zheng Ren, Weidong Yang, Yang Chen, Yingmei Fu, Xiaobei Chen, Jochen WL Cals, and Fengmin Zhang. "Knowledge, attitude and practice of antibiotics: a questionnaire study among 2500 Chinese students." BMC medical education 13, no. 1 (2013): 163.

18. Jamhour, Antoun, Ammar El-Kheir, Pascale Salameh, Pierre Abi Hanna, and Hanine Mansour. "Antibiotic knowledge and self-medication practices in a developing country: A cross-sectional study." American journal of infection control 45, no. 4 (2017): 384-388.

19. Jayabalan, Nalinidevi, Nitya Selvaraj, Suganya Ganesan, Meher Ali Rajamohammad, and Isswariya Anandan. "A questionnaire based survey on knowledge, attitude and behaviour of antibiotic usage and resistance among undergraduates in South Indian teaching hospital." International Journal of Basic & Clinical Pharmacology 7, no. 10 (2018): 1991-1997.

20. June 2019 KFF Health Tracking Poll. (USA)

21. Kamata, Kazuhiro, Yasuharu Tokuda, Yoshiaki Gu, Norio Ohmagari, and Katsunori Yanagihara. "Public knowledge and perception about antimicrobials and antimicrobial resistance in Japan: A national questionnaire survey in 2017." PloS one 13, no. 11 (2018).

22. Kandelaki, Ketevan, Cecilia Stålsby Lundborg, and Gaetano Marrone. "Antibiotic use and resistance: a cross-sectional study exploring knowledge and attitudes among school and institution personnel in Tbilisi, Republic of Georgia." BMC research notes 8, no. 1 (2015): 495.

23. Kim, So Sun, Seongmi Moon, and Eun Jung Kim. "Public knowledge and attitudes regarding antibiotic use in South Korea." Journal of Korean Academy of Nursing 41, no. 6 (2011): 742-749.

24. Larson, Elaine L., Joann Dilone, Magaly Garcia, and Janice Smolowitz. "Factors which influence Latino community members to self-prescribe antibiotics." Nursing research 55, no. 2 (2006): 94-102.

25. Oh, Ai Ling, Mohamed Azmi Hassali, Mahmoud Sadi Al-Haddad, Syed Azhar Syed Sulaiman, Asrul Akmal Shafie, and Ahmed Awaisu. "Public knowledge and attitudes towards antibiotic usage: a cross-sectional study among the general public in the state of Penang, Malaysia." *The Journal of Infection in Developing Countries* 5, no. 05 (2011): 338-347.

25. Lim, Ka Keat, and Chew Charn Teh. "A cross sectional study of public knowledge and attitude towards antibiotics in Putrajaya, Malaysia." Southern med review 5, no. 2 (2012): 26.

26. Mazińska, Beata, Izabela Strużycka, and Waleria Hryniewicz. "Surveys of public knowledge and attitudes with regard to antibiotics in Poland: Did the European Antibiotic Awareness Day campaigns change attitudes?." PloS one 12, no. 2 (2017).

27. McNulty, Cliodna AM, Simon M. Collin, Emily Cooper, Donna M. Lecky, and Chris C. Butler. "Public understanding and use of antibiotics in England: findings from a household survey in 2017." BMJ open 9, no. 10 (2019).

28. Mouhieddine, Tarek H., Zeinab Olleik, Muhieddine M. Itani, Soumayah Kawtharani, Hussein Nassar, Rached Hassoun, Zeinab Houmani et al. "Assessing the Lebanese population for their knowledge, attitudes and practices of antibiotic usage." Journal of infection and public health 8, no. 1 (2015): 20-31.

29. Muras, Magdalena, Jacek Krajewski, Marek Nocun, and Maciek Godycki-Cwirko. "A survey of patient behaviours and beliefs regarding antibiotic self-medication for respiratory tract infections in Poland." Archives of medical science: AMS 9, no. 5 (2013): 854.

30. Norris, Pauline, Marianna Churchward, Fuafiva Fa'alau, and Cecilia Va’ai. "Understanding and use of antibiotics amongst Samoan people in New Zealand." Journal of primary health care 1, no. 1 (2009): 30-35.

31. Panagakou, Sotiria G., Νikos Spyridis, Vassiliki Papaevangelou, Kalliopi M. Theodoridou, Georgia P. Goutziana, Maria N. Theodoridou, George A. Syrogiannopoulos, and Christos S. Hadjichristodoulou. "Antibiotic use for upper respiratory tract infections in children: a cross-sectional survey of knowledge, attitudes, and practices (KAP) of parents in Greece." BMC pediatrics 11, no. 1 (2011): 60.

32. Parimi, Neeta, Lexley M. Pinto Pereira, and Parimi Prabhakar. "The general public's perceptions and use of antimicrobials in Trinidad and Tobago." Revista Panamericana de Salud Pública 12 (2002): 11-18.

33. Pavydė, Eglė, Vincentas Veikutis, Asta Mačiulienė, Vytautas Mačiulis, Kęstutis Petrikonis, and Edgaras Stankevičius. "Public knowledge, beliefs and behavior on antibiotic use and self-medication in Lithuania." International journal of environmental research and public health 12, no. 6 (2015): 7002-7016.

34. Pereko, Dawn D., Martie S. Lubbe, and Sabiha Y. Essack. "Public knowledge, attitudes and behaviour towards antibiotic usage in Windhoek, Namibia." Southern African Journal of Infectious Diseases 30, no. 4 (2015): 134-137.

35. Pew Foundation - 2012. Americans’ Knowledge Of And Attitudes Toward Antibiotic Resistance (USA)

36. Report: General Public's Knowledge, Attitude and Practice Survey on Antimicrobial Resistance 2016/17

37. Sa’ed, H. Zyoud, Adham Abu Taha, Khulood F. Araj, Islam A. Abahri, Ansam F. Sawalha, Waleed M. Sweileh, Rahmat Awang, and Samah W. Al-Jabi. "Parental knowledge, attitudes and practices regarding antibiotic use for acute upper respiratory tract infections in children: a cross-sectional study in Palestine." BMC pediatrics 15, no. 1 (2015): 176.

38. Scaioli, Giacomo, Maria R. Gualano, Renata Gili, Simona Masucci, Fabrizio Bert, and Roberta Siliquini. "Antibiotic use: a cross-sectional survey assessing the knowledge, attitudes and practices amongst students of a school of medicine in Italy." PloS one 10, no. 4 (2015).

39. Shehadeh, Mayadah, Ghadeer Suaifan, Rula M. Darwish, Mayyada Wazaify, Luna Zaru, and Suzan Alja’fari. "Knowledge, attitudes and behavior regarding antibiotics use and misuse among adults in the community of Jordan. A pilot study." Saudi Pharmaceutical Journal 20, no. 2 (2012): 125-133.

40. Sien, Koo Hui, and Marhanis Salihah Omar. "Knowledge and attitude towards antibiotic use and awareness on antibiotic resistance among older people in Malaysia." Proceedings of the Pakistan Academy of Sciences: B. Life and Environmental Sciences 55, no. 2 (2018): 1-9.

41. Sutradhar, Kumar Bishwajit, Anamika Saha, Naz Hasan Huda, and Riaz Uddin. "Irrational use of antibiotics and antibiotic resistance in southern rural Bangladesh: perspectives from both the physicians and patients." Annual Research & Review in Biology (2014): 1421-1430.

42. Togoobaatar, Ganchimeg, Nayu Ikeda, Moazzam Ali, Munkhbayarlakh Sonomjamts, Sarangerel Dashdemberel, Rintaro Mori, and Kenji Shibuya. "Survey of non-prescribed use of antibiotics for children in an urban community in Mongolia." Bulletin of the World Health Organization 88 (2010): 930-936.

43. Tshokey, Tshokey, Deepika Adhikari, Thupten Tshering, Sangay Wangmo, and Kinley Wangdi. "Assessing the knowledge, attitudes, and practices on antibiotics among the general public attending the outpatient pharmacy units of hospitals in Bhutan: a cross-sectional survey." Asia Pacific Journal of Public Health 29, no. 7 (2017): 580-588.

44. Visschers, Vivianne, Vanessa Feck, and Anne Herrmann. "“Contradictio in opinionibus”: The Swiss public’s attitudes and beliefs about antibiotics and antibiotic resistance." (2018).

45. Voidăzan, Septimiu, Geanina Moldovan, Lavinia Voidăzan, Ancuța Zazgyva, and Horațiu Moldovan. "Knowledge, Attitudes And Practices Regarding The Use Of Antibiotics. Study On The General Population Of Mureş County, Romania." Infection and Drug Resistance 12 (2019): 3385.

46. World Health Organization. "Antibiotic resistance: Multi-country public awareness survey." (2015).

47. Yezli, Saber, Yara Yassin, Abdulaziz Mushi, Fuad Maashi, Nibras Aljabri, Gamal Mohamed, Kingsley Bieh, Awam Awam, and Badriah Alotaibi. "Knowledge, attitude and practice (KAP) survey regarding antibiotic use among pilgrims attending the 2015 Hajj mass gathering." Travel medicine and infectious disease 28 (2019): 52-58.

48. Yousif, M., and I. Abubaker. "Prevalence, determinants and practices of self-medication with antibiotics–a population based survey in Taif, Kingdom of Saudi Aarabia ksa." Age 228, no. 172 (2015): 57-50.

49. Zajmi, Drita, Merita Berisha, Ilir Begolli, Rina Hoxha, Rukije Mehmeti, Gjyle Mulliqi-Osmani, Arsim Kurti, Afrim Loku, and Lul Raka. "Public knowledge, attitudes and practices regarding antibiotic use in Kosovo." Pharmacy Practice (Granada) 15, no. 1 (2017).

50. , X., H. Pan, Z. Yang, B. Cui, D. Zhang, and W. Ba-Thein. "Self-medication practices with antibiotics among Chinese university students." Public health 130 (2016): 78-83.

51. Zucco, Rossella, Francesco Lavano, Rosa Anfosso, Aida Bianco, Claudia Pileggi, and Maria Pavia. "Internet and social media use for antibiotic-related information seeking: Findings from a survey among adult population in Italy." International journal of medical informatics 111 (2018): 131-139.

52. El Zowalaty, Mohamed E., Tatiana Belkina, Saleh A. Bahashwan, Ahmed E. El Zowalaty, Jurjen Duintjer Tebbens, Hassan A. Abdel-Salam, Adel I. Khalil et al. "Knowledge, awareness, and attitudes toward antibiotic use and antimicrobial resistance among Saudi population." *International journal of clinical pharmacy* 38, no. 5 (2016): 1261-1268.

53. World Health Organization. "Antibiotic resistance: Multi-country public awareness survey." (2015).

54. Azevedo, Maria Manuel, Céline Pinheiro, John Yaphe, and Fátima Baltazar. "Portuguese students' knowledge of antibiotics: a cross-sectional study of secondary school and university students in Braga." *BMC Public Health* 9, no. 1 (2009): 359.

55. Ramchurren, K., Y. Balakrishna, and S. Mahomed. "Patients’ knowledge, attitudes and practices regarding antibiotic use at a regional hospital in KwaZulu-Natal, South Africa 2017." *Southern African Journal of Infectious Diseases* (2018): 1-6.

56. Saha, Moni Rani, Shammy Sarwar, Manik Chandra Shill, and Mohammad Shahriar. "Patients' Knowledge and Awareness towards Use of Antibiotics in Bangladesh: A Cross-sectional Study Conducted in Three Tertiary Healthcare Centers in Bangladesh." *Stamford Journal of Pharmaceutical Sciences* 3, no. 1 (2010): 54-58.

57. Cheng, Jing, Caroline Coope, Jing Chai, Isabel Oliver, Anthony Kessel, Debin Wang, and Yehuan Sun. "Knowledge and behaviors in relation to antibiotic use among rural residents in Anhui, China." *Pharmacoepidemiology and drug safety* 27, no. 6 (2018): 652-659.

58. Jifar, ALemnesh, and Yohanes Ayele. "Assessment of Knowledge, Attitude, and Practice toward Antibiotic Use among Harar City and Its Surrounding Community, Eastern Ethiopia." *Interdisciplinary perspectives on infectious diseases* 2018 (2018).

59. Salm, Florian, Clemens Ernsting, Adelheid Kuhlmey, Melanie Kanzler, Petra Gastmeier, and Paul Gellert. "Antibiotic use, knowledge and health literacy among the general population in Berlin, Germany and its surrounding rural areas." *PloS one* 13, no. 2 (2018).

60. Raupach-Rosin, Heike, Nicole Rübsamen, Gesa Schütte, Gabriele Raschpichler, Pa Saidou Chaw, and Rafael Mikolajczyk. "Knowledge on antibiotic use, self-reported adherence to antibiotic intake, and knowledge on multi-drug resistant pathogens-results of a population-based survey in Lower Saxony, Germany." *Frontiers in microbiology* 10 (2019): 776.

61. Gupta, Kishan Chand. "Knowledge, attitude and practice of parents towards antibiotic usage in children." *Journal of Advanced Medical and Dental Sciences Research* 7, no. 11 (2019): 80-83.

62. Godman, Brian, Joseph Fadare, Dan Kibuule, Lyna Irawati, Mwangana Mubita, Olayinka Ogunleye, Margaret Oluka et al. "Initiatives across countries to reduce antibiotic utilisation and resistance patterns: impact and implications." In *Drug resistance in bacteria, fungi, malaria, and cancer*, pp. 539-576. Springer, Cham, 2017.

63. Prigitano, Anna, Luisa Romanò, Francesco Auxilia, Silvana Castaldi, and Anna M. Tortorano. "Antibiotic resistance: Italian awareness survey 2016." *Journal of infection and public health* 11, no. 1 (2018): 30-34.

64. Odetokun, Ismail A., Uduak Akpabio, Nma B. Alhaji, Khalid T. Biobaku, Nurudeen O. Oloso, Ibraheem Ghali-Mohammed, Asmau J. Biobaku, Victoria O. Adetunji, and Folorunso O. Fasina. "Knowledge of Antimicrobial Resistance among Veterinary Students and Their Personal Antibiotic Use Practices: A National Cross-Sectional Survey." *Antibiotics* 8, no. 4 (2019): 243.

65. Waaseth, Marit, Abdifatah Adan, Ingrid L. Røen, Karoline Eriksen, Tijana Stanojevic, Kjell H. Halvorsen, Beate H. Garcia et al. "Knowledge of antibiotics and antibiotic resistance among Norwegian pharmacy customers–a cross-sectional study." *BMC public health* 19, no. 1 (2019): 66.

66. Demoré, Béatrice, Lucie Mangin, Gianpiero Tebano, Céline Pulcini, and Nathalie Thilly. "Public knowledge and behaviours concerning antibiotic use and resistance in France: a cross-sectional survey." *Infection* 45, no. 4 (2017): 513-520.

67. Almohammed, Rimah A., and Emma L. Bird. "Public knowledge and behaviours relating to antibiotic use in Gulf Cooperation Council countries: A systematic review." *Journal of infection and public health* 12, no. 2 (2019): 159-166.

68. Uddin, Md Najem, Md Hafizur Rahman, Md Mehedi Hasan, Md Nazmul Islam, Md Bokhtear Sarkar, Md Rafiul Islam, SM Abdul Barik, and Md Nazmul Sardar. "Survey on Antimicrobial Resistance: Reason behind the Misuse of Antibiotics in Bangladesh." *Journal of Pharmaceutical Research International* (2017): 1-8.

69. Pavydė, Eglė, Vincentas Veikutis, Asta Mačiulienė, Vytautas Mačiulis, Kęstutis Petrikonis, and Edgaras Stankevičius. "Public knowledge, beliefs and behavior on antibiotic use and self-medication in Lithuania." *International journal of environmental research and public health* 12, no. 6 (2015): 7002-7016.

70. Atif, Muhammad, Saima Asghar, Irem Mushtaq, Iram Malik, Anum Amin, Zaheer-Ud-Din Babar, and Shane Scahill. "What drives inappropriate use of antibiotics? A mixed methods study from Bahawalpur, Pakistan." *Infection and drug resistance* 12 (2019): 687.

71. Lyimo, Sarah R., Geoffrey N. Sigalla, Basiliana Emidi, Maseke R. Mgabo, and Debora C. Kajeguka. "Cross-sectional Survey on Antibiotic Prescription Practices Among Health Care Providers in Rombo District, Northern Tanzania." *EA Health Research Journal* 2, no. 1 (2018): 10-17.

72. Widayati, Aris, Sri Suryawati, Charlotte De Crespigny, and Janet E. Hiller. "Knowledge and beliefs about antibiotics among people in Yogyakarta City Indonesia: a cross sectional population-based survey." *Antimicrobial resistance and infection control* 1, no. 1 (2012): 38.

73. McNulty, Cliodna AM, Simon M. Collin, Emily Cooper, Donna M. Lecky, and Chris C. Butler. "Public understanding and use of antibiotics in England: findings from a household survey in 2017." *BMJ open* 9, no. 10 (2019).

74. de Bont, Eefje GPM, Nick A. Francis, Geert-Jan Dinant, and Jochen WL Cals. "Parents’ knowledge, attitudes, and practice in childhood fever: an internet-based survey." *Br J Gen Pract* 64, no. 618 (2014): e10-e16.

75. Eng, Jodi Vanden, Ruthanne Marcus, James L. Hadler, Beth Imhoff, Duc J. Vugia, Paul R. Cieslak, Elizabeth Zell et al. "Consumer attitudes and use of antibiotics." *Emerging infectious diseases* 9, no. 9 (2003): 1128.

76. Gül, Serdar, Doğan Barış Öztürk, Muhittin Serkan Yılmaz, and Esen Uz Gül. "Evaluation of public knowledge and attitudes regarding self medication with antibiotics in Ankara." *Turkish Bulletin of Hygiene and Experimental Biology* 71, no. 3 (2014): 107-112.

76. Baş, Muhammed Kağan, Fatima Betul Basturk, and Hesna SAZAK ÖVECOGLU. "Awareness of Antibiotics and Analgesics Use In Marmara University Hospital." *International Journal of Scientific Research in Dental and Medical Sciences* 1, no. 4 (2019): 57-61.

77. Auta, Asa, Samuel B. Banwat, Shalkur David, Dauda A. Dangiwa, Esther Ogbole, and Amom J. Tor-anyiin. "Antibiotic use in some Nigerian communities: knowledge and attitudes of consumers." Tropical Journal of Pharmaceutical Research 12, no. 6 (2013): 1087-1092.

78. Bert, Fabrizio, Maria R. Gualano, Renata Gili, Giacomo Scaioli, Emanuela Lovato, Italo F. Angelillo, Silvio Brusaferro et al. "Knowledge and attitudes towards the use of antibiotics in the paediatric age group: a multicenter survey in Italy." The European Journal of Public Health 27, no. 3 (2017): 506-512.

79. Gonzales, Ralph, Alma Ethelia López-Caudana, Tulia González-Flores, Janaki Jayanthan, Kitty K. Corbett, and Hortensia Reyes-Morales. "Antibiotic knowledge and self-care for acute respiratory tract infections in Mexico." salud pública de méxico 54, no. 2 (2012): 152-157.

80. Inácio, João, Lara-Marie Barnes, Simon Jeffs, Patrícia Castanheira, Myra Wiseman, Sónia Inácio, Lucas Bowler, and Alison Lansley. "Master of Pharmacy students’ knowledge and awareness of antibiotic use, resistance and stewardship." Currents in Pharmacy Teaching and Learning 9, no. 4 (2017): 551-559.

81. Jayabalan, Nalinidevi, Nitya Selvaraj, Suganya Ganesan, Meher Ali Rajamohammad, and Isswariya Anandan. "A questionnaire based survey on knowledge, attitude and behaviour of antibiotic usage and resistance among undergraduates in South Indian teaching hospital." International Journal of Basic & Clinical Pharmacology 7, no. 10 (2018): 1991-1997.

82. Lim, Ka Keat, and Chew Charn Teh. "A cross sectional study of public knowledge and attitude towards antibiotics in Putrajaya, Malaysia." Southern med review 5, no. 2 (2012): 26.

83. Mbwambo, Goodluck, Basiliana Emidi, Maseke R. Mgabo, Geofrey Nimrod Sigalla, and Debora C. Kajeguka. "Community knowledge and attitudes on antibiotic use in Moshi Urban, Northern Tanzania: Findings from a cross sectional study." African Journal of Microbiology Research 11, no. 25 (2017): 1018-1026.

84. Mouhieddine, Tarek H., Zeinab Olleik, Muhieddine M. Itani, Soumayah Kawtharani, Hussein Nassar, Rached Hassoun, Zeinab Houmani et al. "Assessing the Lebanese population for their knowledge, attitudes and practices of antibiotic usage." Journal of infection and public health 8, no. 1 (2015): 20-31.

85. Nepal, Anant, Delia Hendrie, Suzanne Robinson, and Linda A. Selvey. "Knowledge, attitudes and practices relating to antibiotic use among community members of the Rupandehi District in Nepal." BMC public health 19, no. 1 (2019): 1558.

86. Norris, Pauline, Lye Funn Ng, Victoria Kershaw, Fady Hanna, Angela Wong, Meghna Talekar, Jin Oh, Maryam Azer, and Lynn Cheong. "Knowledge and reported use of antibiotics amongst immigrant ethnic groups in New Zealand." Journal of Immigrant and Minority Health 12, no. 1 (2010): 107.

87. Parimi, Neeta, Lexley M. Pinto Pereira, and Parimi Prabhakar. "The general public's perceptions and use of antimicrobials in Trinidad and Tobago." Revista Panamericana de Salud Pública 12 (2002): 11-18.

88. Rábano-Blanco, Andrea, Eva María Domínguez-Martís, Diego Gabriel Mosteiro-Miguéns, Manuel Freire-Garabal, and Silvia Novío. "Nursing Students’ Knowledge and Awareness of Antibiotic Use, Resistance and Stewardship: A Descriptive Cross-Sectional Study." Antibiotics 8, no. 4 (2019): 203.

89. Ramchurren, K., Y. Balakrishna, and S. Mahomed. "Patients’ knowledge, attitudes and practices regarding antibiotic use at a regional hospital in KwaZulu-Natal, South Africa 2017." Southern African Journal of Infectious Diseases (2018): 1-6.

90. Raupach-Rosin, Heike, Nicole Rübsamen, Gesa Schütte, Gabriele Raschpichler, Pa Saidou Chaw, and Rafael Mikolajczyk. "Knowledge on antibiotic use, self-reported adherence to antibiotic intake, and knowledge on multi-drug resistant pathogens-results of a population-based survey in Lower Saxony, Germany." Frontiers in microbiology 10 (2019): 776.

91. Sien, Koo Hui, and Marhanis Salihah Omar. "Knowledge and attitude towards antibiotic use and awareness on antibiotic resistance among older people in Malaysia." Proceedings of the Pakistan Academy of Sciences: B. Life and Environmental Sciences 55, no. 2 (2018): 1-9.

92. Tadvi, Naser Ashraf, Hamad Abdulaziz Al Olah, Abdallah Bejad AlMjlad, Mazin Talal Al Shammari, Mishary Malik Al Shammari, Sajid Hussain, and Waqas Sami. "Knowledge, Attitudes and Practice Regarding Antimicrobial Resistance and Antimicrobial Use among Undergraduate Medical Students in Majmaah, Saudi Arabia." Journal of Research in Medical and Dental Science 7, no. 2 (2019): 75-81.

93. Tshokey, Tshokey, Deepika Adhikari, Thupten Tshering, Sangay Wangmo, and Kinley Wangdi. "Assessing the knowledge, attitudes, and practices on antibiotics among the general public attending the outpatient pharmacy units of hospitals in Bhutan: a cross-sectional survey." Asia Pacific Journal of Public Health 29, no. 7 (2017): 580-588.

94. Zaidi, Syed Faisal, Rakan Alotaibi, Abdulaziz Nagro, Muath Alsalmi, Hidaya Almansouri, Muhammad Anwar Khan, Aslam Khan, and Ismail Memon. "Knowledge and Attitude Towards Antibiotic Usage: A Questionnaire-Based Survey Among Pre-Professional Students at King Saud bin Abdulaziz University for Health Sciences on Jeddah Campus, Saudi Arabia." Pharmacy 8, no. 1 (2020): 5.
