# Supplementary material for: A natural history museum visitor survey of perception, attitude and knowledge (PAK) of microbes and antibiotics
Source: PLoS One. 2021 Sep 22;16(9):e0257085. doi: 10.1371/journal.pone.0257085 (PMC8457478; doi:10.1371/journal.pone.0257085)
Supplement: S2 File — (DOCX) [file pone.0257085.s002.docx]

**Supplemental File 2**

**Kiosk Design**

The physical dimensions of the kiosk (48” height, 24” depth, 24” width with a tilted 27” screen; **Figure S2.1**) were designed to allow its easy movement and nonintrusive placement in existing halls at the AMNH. The kiosk was stationed on a Mac mini with the specs being 2.6GHz dual-core Intel Core i5 Turbo Boost (with up to 3.1GHz) and a 3MB on-chip shared L3 cache 8GB of 1600MHz LPDDR3 memory. The display was a 27" ELO touch screen. HTML, javascript and CSS were used to custom write the software, including some animations written in p5.js which is a creative-coding framework for javascript. The data were stored in a MongoDB database and synced to the cloud using Dropbox to allow review of the data without repeatedly visiting the kiosk. The web app produced a real time archive of answers and posted a summary in several formats at least once a week. The app we used and all of the data archives are available upon request or are online (<https://www.amnh.org/research/staff-directory/robert-desalle>) under “SEPA kiosk”.

**Website Description**

The website can be found at <https://www.amnh.org/research/staff-directory/robert-desalle> under “SEPA kiosk” and has a simple to navigate homepage (**Figure S2.2**). The homepage has three main menus - “General AMNH/SEPA Information”, “Survey Questions” and “SEPA kiosk Results”. The SEPA kiosk results menu contains four submenus – 1. The kiosk and its description; 2. A global map showing the number of respondents by country for the two surveys (**Figure S2.3**); 3. An analysis of the basic demographics of the two surveys; 4. A note on the use of English for the surveys. The global map mentioned above allows researchers to rapidly examine the sample sizes for different countries involved in the surveys. The note on English usage in the surveys was conducted to demonstrate the effect of English usage on results obtained in the two surveys. The general results of this analysis suggest a slight effect, but in general we conclude that the results of the surveys are a valid first look at the attitudes and knowledge of people globally on issues related to microbes. We also point out that the use of the kiosk was voluntary and most people regardless of language of origin interacted with it because they had a working familiarity with English. The download area for the data sets can be found below the three main menus on the website.

**A note on the general utility of the AMNH-SEPA surveys**

We have made all of the raw data available to the general public for download (see (<https://www.amnh.org/research/staff-directory/robert-desalle> under “SEPA kiosk”). We point out that several other topics are addressed in the surveys and could be used by researchers interested in handwashing behaviors, use of sanitizers, probiotic use and general hygienic behavior of people. We do recognize that for most of the countries sample sizes are relatively small, which is why we stratified by general geographic region in the analyses presented in this paper. There are seventeen and nineteen individual countries with N>95 and most of these are North American, European and large developed Asian countries, but if the threshold for minimal sample size is lowered to 45, the sample size doubles and begins to include many smaller Asian countries, several African countries and middle eastern countries.

There are three major reasons for releasing these data for download to the research community. First, we hope that by making these data available more detailed analyses can be accomplished by interested researchers who have stakes in gender-based, age-based and nationality-based educational evaluation and research. The data are downloadable in easy-to-manipulate .cvs and excel files and parsing of data in this format is simple. Second, we suggest that the availability of these data can serve as a baseline for future surveys about knowledge of microbes, antibiotics, probiotics and hand sanitizers. The data base for the most part is sufficiently large and the questions sufficiently basic to allow for more detailed and involved questions to be posed in future surveys. Third, we believe that the data base and analysis herein demonstrate the utility of kiosk-based survey tools when used in a museum or science center. Visitors to these institutions make good survey subjects as they often have an established interest in science and our results indicate that many are comfortable in initiating and completing surveys. However, we offer the important caveat that museum and science center visitors are a somewhat more mobile and better-informed section of the population than others. Importantly, visitors can undertake kiosk surveys free of privacy concerns often associated with online surveys and within an institution that already enjoys high levels of public trust (Griffith and King, 2005; Leiserowitz,et al 2010; Skorton 2018; Schneider 2017). Kiosk surveys may also be more trusted regardless of location. For example, the few published comparisons (mostly in the medical realm; Jones, 2009; Hankin et al., 2015) of kiosk versus other surveys reveal that people are more likely to disclose alcoholism/addiction and other important personal data when interacting with a kiosk than in an in-person interviews. We further suggest that visitor survey kiosks, coupled with the growing international community of science centers (e. g. Association of Science-Technology Centers - [ASTC.org](https://nam04.safelinks.protection.outlook.com/?url=http%3A%2F%2FASTC.org&data=02%7C01%7Cdesalle%40amnh.org%7Ca1ba7f547af246bfb4a608d708421c4f%7Cbe0003e8c6b9496883aeb34586974b76%7C0%7C0%7C636986952361332172&sdata=tNoVZnVJcUNAytXx10qOZIMQ0CP26D%2BVpOJMydP8jFs%3D&reserved=0)) where a kiosk system could easily be installed in several institutions simultaneously, offer an opportunity to create a global infrastructure, supporting national and internationally coordinated surveys.  Such surveys, particularly if addressing issues of national or global significance may lead to better understanding of public attitudes and potentially to evaluate the impact of education programs.

**Utility and accessibility of the website**

The website is linked to the American Museum of Naturel History’s web system. The AMNH is a longstanding collections-based institution and its capacity for web traffic is well established. Maintenance of the website is conducted by the internal IT system at the AMNH. The AMNH website has been a mainstay in informal education websites for the past thirty years and the potential for its maintenance into the future is not in question. As we point out in the discussion of this paper, the use of kiosks as sources of primary data for STEM based surveys will only increase in the future. This website and the methods we have used to highlight the surveys and make them available for other researchers serves as a template for future efforts in this area. We envision that the current SEPA kiosk website can easily be incorporated into a larger web system that will host the accessibility of kiosk survey data to the research community.


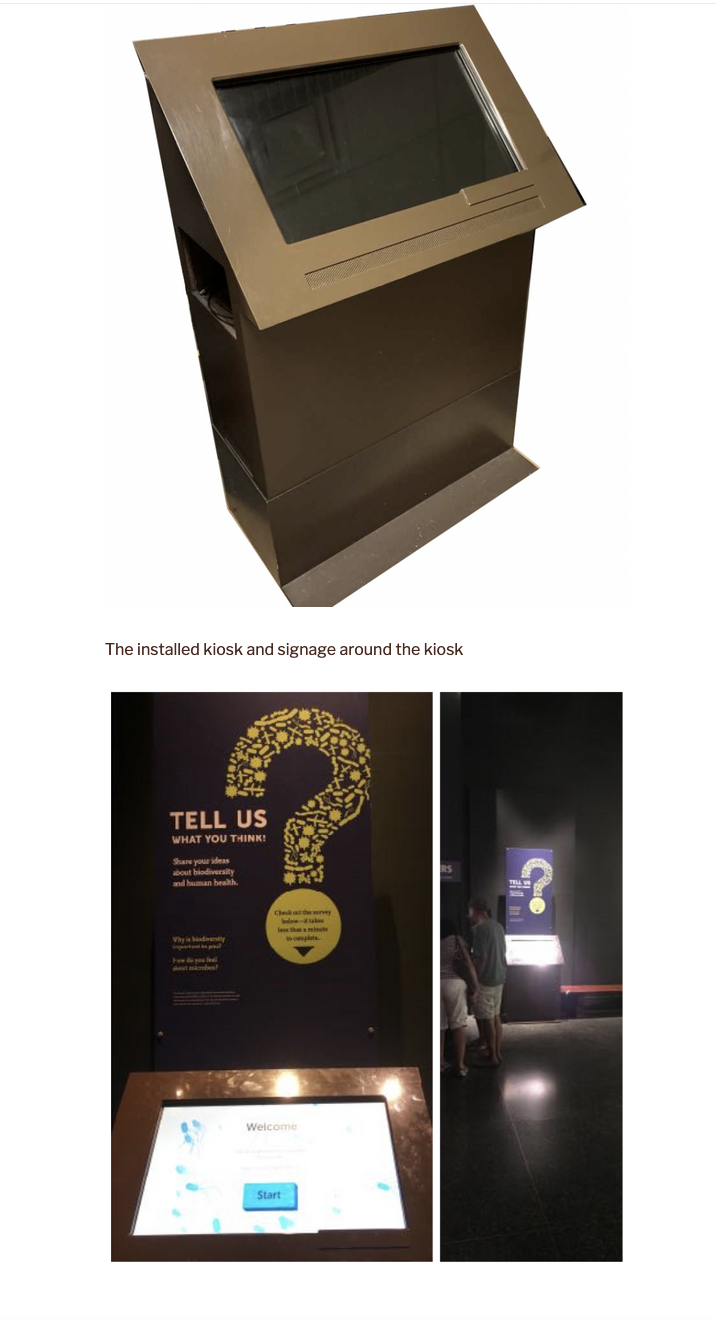


Supplemental File 2 – Figure S2.1: **The installed kiosk and signage around it.** The kiosk was designed with appealing graphics and was placed in a prominent position with prominent signage around it in the AMNH’s Hall of Biodiversity. Its dimensions and specs are described in the text.


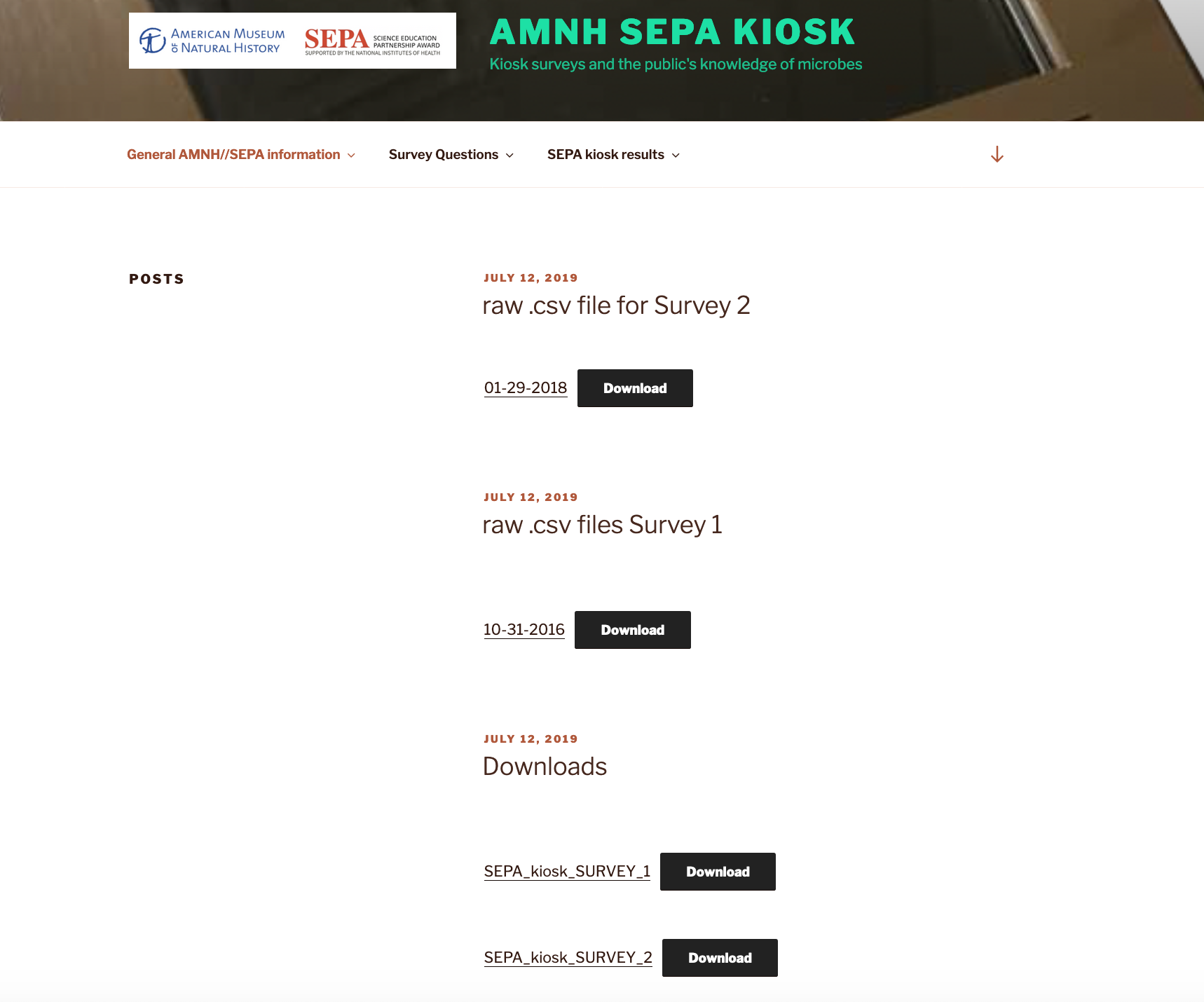


Supplemental File 2 – Figure S2.2: **Home page showing the three menus for the website.** The first of the three menus is “General AMNH/SEPA Information” under which four submenu items are available – 1. AMNH Mission Statement; 2. NIH SEPA information; 3. The AMNH/SEPA kiosk advisory council information; 4. Links to AMNH SEPA Science Café and Teen Science Café (two related products in the SEPA project). The second menu is “Survey Questions” under which three submenus are available 1. The SEPA polling rationale; 2. The 2017 SEPA poll questions; 3. The 2018 SEPA poll questions. The third menu entitled “SEPA kiosk Results” includes several submenus discussed in the text.

**References**

Griffith and King 2005. <http://www.interconnectionsreport.org/reports/IMLSMusRpt20080312kjm.pdf>

Hankin, Abigail, Leon Haley, Amy Baugher, Kia Colbert, and Debra Houry. "Kiosk versus in-person screening for alcohol and drug use in the emergency department: patient preferences and disclosure." Western journal of emergency medicine 16, no. 2 (2015): 220.

Jones, Ray. "The role of health kiosks in 2009: literature and informant review." International journal of environmental research and public health 6, no. 6 (2009): 1818-1855.

Leiserowitz, A., Smith, N. & Marlon, J.R. (2010) Americans’ Knowledge of Climate Change. Yale University. New Haven, CT: Yale Project on Climate Change Communication. http://environment.yale.edu/climate/files/ClimateChangeKnowledge2010.pdf

Schneider, M. (2017). People Trust Museums More Than Newspapers. Here Is Why That Matters Right Now (DATA). <https://www.colleendilen.com/2017/04/26/people-trust-museums-more-than-newspapers-here-is-why-that-matters-right-now-data/>

Skorton, 2018. Americans’ Knowledge of Climate Change.

<https://environment.yale.edu/climate-communication-OFF/files/ClimateChangeKnowledge2010.pdf>
