# Supplementary material for: A natural history museum visitor survey of perception, attitude and knowledge (PAK) of microbes and antibiotics
Source: PLoS One. 2021 Sep 22;16(9):e0257085. doi: 10.1371/journal.pone.0257085 (PMC8457478; doi:10.1371/journal.pone.0257085)
Supplement: S3 File — (DOCX) [file pone.0257085.s003.docx]

**Supplemental File 3. Assessing the impact of non-native English speakers on results**

Since a large number of the respondents are non-native English speakers we attempted to assess the impact of language on results of the surveys. The first hypothesis in Table 2 addresses how the use of English in the surveys might impact the results. There are many ways to assess this impact and we chose a straight comparative approach. We used Survey 1/Question 1 (“Which words come to mind when you hear the word microbe?"), which had six possible answers as choices (germ, disease, tiny, essential, beneficial and biodiversity) and any number of answers could be chosen by the respondent. Two response choices are negative characterizations of microbes (germ, disease). Three response choices are positive characterizations of microbes (essential, beneficial, biodiversity) and one response choice was a neutral characterization of microbes (tiny). We reasoned that extreme differences in patterns of microbe characterization between native English speaking and non-native English-speaking respondents would indicate an impact of language on survey results. We therefore tested the frequency of four different combinations of words comb1= beneficial + essential + biodiversity (all positive characterizations); comb2= any combination of terms that omits all three positive characterizations (omit beneficial + essential and biodiversity); comb3 = all six positive, negative, and neutral characterizations used in one answer; and comb4 = germ + disease + tiny (all negative or neutral characterizations). **Supplemental** **Figure 1.1** shows the results of this analysis. The data show that there are small but non-significant differences between the respondents from the four countries.

**Supplemental Figure 3.1. Frequencies of word combinations in response to Survey 1/ Question 1 in residents of four countries**. The categories in the graph are **Comb1**= beneficial + essential + biodiversity (all positive characterizations); **Comb2**= any combination of terms that omits all three positive characterizations (omit beneficial + essential and biodiversity); **Comb3** = all six positive, negative, and neutral characterizations used in one answer; and **Comb4** = germ + disease + tiny (all negative or neutral characterizations).

We also used Survey 1/Question 2 to compare survey responses across native and non-native English speakers (“Which of these is an antibiotic? Select as many as you like!"). There are five possible answer choices (aspirin, Tylenol, Valium, penicillin and Benadryl) for this question and any combination of the five could be selected by the respondent. In addition to the generic names listed above, we also indicated common commercial names for these compounds. Valium was listed as “Diazepam (e.g. Valium)” to give the respondent another way to identify the compound. The single correct answer to this question is “penicillin”, the only antibiotic in the list. The results of this analysis (**Figure 3.2**) suggest that English usage may slightly impact responses.

**Supplemental Figure 3.2. Percent of correct answers to Survey 1/Question 3 by country of the respondents.** The only correct answer was penicillin alone; if a respondent indicated that other medicines were antibiotics, then their response was considered to be incorrect, even when penicillin was selected. (AU=Australia, US=United States of America, JP=Japan and CH=China). The disparity in correct responses between native English and non-native English speakers ranges from 10% (US compared to AU; significant at p<0.05 using Fisher Exact test) to 20% (JP compared to US; significant at p<0.05 using Fisher Exact test). These analyses also reveal greater variation related to more technical terms like compound names, but less with usage of descriptive words (**Supplemental** **Figure 1.1**).

Analyses of responses to these two questions indicate a greater discrepancy in native versus non-native English speaker responses when medical compound names are used (**Supplemental** **Figure 3.2**) than when descriptive terms are used (**Supplemental** **Figure 3.1**). As such, we report an upper and lower boundary on the frequency of incorrect responses to the survey questions. We suggest that in general regardless of the language of respondents the answers are informative of general trends of perceptions, attitudes and knowledge (PAK). However, to account for possible language effects, we report averages for the frequency of particular responses as ranges to provide estimates of the potential impact of language on the overall conclusions of the study. Finally it is safe to assume that visitors who properly interact with the kiosk are ones who speak and understand English. Non-native speakers more than likely walk away from the kiosk if there are language difficulties.
