# Supplementary material for: A natural history museum visitor survey of perception, attitude and knowledge (PAK) of microbes and antibiotics
Source: PLoS One. 2021 Sep 22;16(9):e0257085. doi: 10.1371/journal.pone.0257085 (PMC8457478; doi:10.1371/journal.pone.0257085)
Supplement: Attachment — Submitted filename: Reponse to reviewers2.docx [file pone.0257085.s004.docx]

Below we address individually all of the comments of the reviewers. Our response to the critiques are colored yellow for R1 and blue for R2

**Reviewer #1:** Concerning the problem of microbial resistances, the studies about general public perception, attitude and Knowledge of microbes and antibiotics are important for the development of interventions to improve this important public health issue. However, I have some concerns about the methodology and the organization of this manuscript.

The last paragraph of the introduction should be modified and include the clear objective of the work. What does the authors means with “The second concerns general attitudes toward microbes, a topic which has not been well surveyed."? There are published studies about it. Did the authors made any review about the quality of published studies?

We have rewritten the introduction to make the objective of the work clearer. We have removed narrative about kiosks and their utility.

While there are a large number of studies using surveys of the PAK of antibiotics and microbial resistance there are as we stated in the manuscript few are focused on how the public perceives microbes (ie. As useful, disgusting, disease causing etc). The ratio in the literature is about 1 to 10 (microbes focused versus antibiotic focused). Many of the survey papers concern medical student attitudes and perceptions. We cannot find strong evidence for the existence of a large literature of PAK on microbes. We have clarified this in our manuscript and added the following references. Again there are hundreds of survey based articles that address PAK to antibiotics and resistance but only ten or so addressing PAK of microbes in general.

A literature search for public understanding of microbes over the past decade gives the following references, four of which are actual surveys and three

(Yeo et al., 2019; Kurt, 2013; McNeil et al., 2010; Loo et al., 2016; Hamdiyati et al., 2017, 2018; Jones et al., 2013; Beovic et al., 2019; Morgan, 1995)

Yeo, Sara K., Ye Sun, Meaghan McKasy, and Erika C. Shugart. "Disgusting microbes: The effect of disgust on perceptions of risks related to modifying microbiomes." *Public Understanding of Science* 28, no. 4 (2019): 433-448.

Kurt, Hakan. "Turkish Student Biology Teachers' Conceptual Structures And Semantic Attitudes Towards Microbes." *Journal of Baltic Science Education* 12, no. 5 (2013): 608.

McNeil, J. N., P-A. Cotnoir, T. Leroux, R. Laprade, and J-L. Schwartz. "A Canadian national survey on the public perception of biological control." *BioControl* 55, no. 4 (2010): 445-454.

Loo, Matthew, Helen Heacock, and Reza Afshari. "Evaluation of the public’s knowledge, attitude, and practice on seafood contaminants." *BCIT Environmental Health Journal* (2016).

Hamdiyati, Y., F. Sudargo, S. Redjeki, and A. Fitriani. "Biology students’ initial mental model about microorganism." In *Journal of Physics, Conference*, vol. 812, no. 1, p. 012027. 2017.

Hamdiyati, Yanti, Fransisca Sudargo, Sri Redjeki, and Any Fitriani. "CHANGING OF STUDENT'S MENTAL MODEL ABOUT VIRUS THROUGH MICROBIOLOGY COURSE PROGRAM BASED ON MENTAL MODEL." *EDUSAINS* 10, no. 1 (2018): 74-82.

Jones, Gail, Grant E. Gardner, Tammy Lee, Kayla Poland, and Sarah Robert. "The impact of microbiology instruction on students’ perceptions of risks related to microbial illness." *International Journal of Science Education, Part B* 3, no. 3 (2013): 199-213.

Beović, Bojana, May Doušak, Céline Pulcini, Guillaume Béraud, Jose Ramon Paño Pardo, David Sánchez-Fabra, Diamantis Kofteridis et al. "Young doctors’ perspectives on antibiotic use and resistance: a multinational and inter-specialty cross-sectional European Society of Clinical Microbiology and Infectious Diseases (ESCMID) survey." *Journal of Antimicrobial Chemotherapy* 74, no. 12 (2019): 3611-3618.

Morgan, M. S. "Perceptions of a medical microbiology service: a survey of laboratory users." *Journal of clinical pathology* 48, no. 10 (1995): 915-918.

More importantly, both reviewers were worried that our paper was stuck between being about kiosk surveys and about attitudes and knowledge of people. We fully admit that we attempted to incorporate both and balance both. It appears though that the reviewers are more interested in the survey attitudes than in the utility of kiosks as a survey tool. So we have revised the manuscript with this in mind.

Please review the lack of references for some statements.

We imagine that this comment is based on this reviewer’s suggestion that surveys about microbes are aplenty in the literature. We hope we have explained that misunderstanding. As for other statements, whenever we have made a claim about sampling or visitor attitudes we have reassessed the statements with respect to the literature and added references to shore up the statement. We have added over thrity references to our literature cited section and there are close to 100 references in Supplemental File 1.

I have some doubts about the sample bias of the kiosk surveys. Participants are not representative of the population ... A kiosk survey in a museum have many bias, because the population are not representative. The cultural interests of the respondents are very similar ... The results cannot be generalized to the general population.
The survey was applied during the same time educational interventions are conducted in the Museum? This another sampling bias! This should be discussed in the Discussion section.

We have added a **limitations** section to the discussion. It now discusses museum goer bias, kiosk bias and language bias.

We cite the following literature with respect to this criticism:

Schneider, M. (2017). People Trust Museums More Than Newspapers. Here Is Why That Matters Right Now (DATA). <https://www.colleendilen.com/2017/04/26/people-trust-museums-more-than-newspapers-here-is-why-that-matters-right-now-data/>

Gunther, Charles F. "Museum-goers: life-styles and learning characteristics." *The educational role of the museum* 2 (1999): 118-130.

Kirchberg, Volker. "Museum visitors and non-visitors in Germany: A representative survey." *Poetics* 24, no. 2-4 (1996): 239-258.

Dickenson, Victoria. "Museum visitor surveys: an overview, 1930–1990." In *Cultural Economics*, pp. 141-150. Springer, Berlin, Heidelberg, 1992.

Marty, Paul F. "Museum websites and museum visitors: Before and after the museum visit." *Museum management and curatorship* 22, no. 4 (2007): 337-360.

Hein, George E. "Museum education." *A companion to museum studies* (2006): 340-352.

John H. Falk, Mark D. Needham. **Measuring the impact of a science center on its community**. Journal of Research in Science Teaching, 2011; 48 (1): 1 DOI: [10.1002/tea.20394](http://dx.doi.org/10.1002/tea.20394)

Falk, John H., and Lynn D. Dierking. *The museum experience revisited*. Routledge, 2016.

Falk, John H., and Mark D. Needham. "Factors contributing to adult knowledge of science and technology." *Journal of Research in Science Teaching* 50, no. 4 (2013): 431-452.

Şentürk, Eray, and Ömer Faruk Özdemir. "The effect of science centres on students' attitudes towards science." *International Journal of Science Education, Part B* 4, no. 1 (2014): 1-24.

Falk, John H., Mark D. Needham, Lynn D. Dierking, and Lisa Prendergast. "International science centre impact study." *Final report. John. H. Falk Research, Corvallis, Oregon* 45 (2014).

Falk, John H., Lynn D. Dierking, Lisa Prendergast Swanger, Nancy Staus, Mariana Back, Chantal Barriault, Carlos Catalao et al. "Correlating science center use with adult science literacy: An international, cross‐institutional study." *Science Education* 100, no. 5 (2016): 849-876.

Suter, Larry E. "Visiting science museums during middle and high school: A longitudinal analysis of student performance in science." *Science Education* 98, no. 5 (2014): 815-839.

Hammerness, Karen, Anna MacPherson, and Preeti Gupta. "Developing a research agenda aimed at understanding the teaching and learning of science at a natural history museum." *Curator: The Museum Journal* 59, no. 4 (2016): 353-367.

We have addressed this problem in the revised manuscript and fully understand what both this reviewer and R2 are saying about the sample not being a true “global” sample, but rather a “museum goer” sample. We hope that we have cleared up this sample description problem in the current version of the manuscript.

The method section should be described more objectively and clearly. For example: In the "survey design" sub-section authors discuss problems about language barriers when surveying. This is not adequate for a methods section. In this section, authors should describe what they do and not what was challenging or the problems they found to do it. All the procedures should be well described for permitting the replication of the study.
“We assessed the impact of native versus nonnative English speakers for some of the questions and suggest that the bias is minimal (see below)” – Below where? In the results section? How was assessed the impact? The methodology used should be described in the methods section! The result of this assessment “bias is minimum” is information for results section!

We have moved the discussion of language bias to the methods section, the results of the analysis to the results section and the details of the analysis to a supplemental file.

Reference to the results during the methods section is not correct! Among other examples, please remove from methods section “see results below”, and references to tables with results.

We have cleaned up this aspect of the paper

The use of medicines brand names, could bias the different response between participants from different countries, because in some countries de drugs could have different brand names.

Yes this is a major point of contention. We have addressed it in the revised version of the manuscript, by pointing out that even if there are some responses dependent on brand names, this does not change the overall result that our museum goers have skewed and incorrect attitudes and knowledge of antibiotics. We also discuss this issue in the “Limitations” section of the paper.

“The respondents’ age and sex are given in Table 2. Most surveys omit children from reporting of results, however for our surveys we retained data for respondents <13 years as the answers for this age did not vary significantly from older respondents.” How did the authors analysed that significance? Answers from 6-years old children were also included?

We cannot assess if answers from 6 year olds were in the sample (as the cutoff was 13 or younger). We recognize that this is problematic. We have however, made clear in the manuscript that there are results from participants in the <13 yrs of age category and briefly report that this age group did not differ significantly from other age groups.

The results section should only contain information about the results and not the procedures for obtaining the data. Descriptions about the methodology used to asses “languages difference” should be moved from results section to the methods section. Also the same for the procedures used to assess attitudes and knowledge.

WE have made these alterations to the M&M section.

Why did the authors did not use data from all the respondents to asses languages differences? Why only two countries that are predominantly non-native English-speaking? To eliminate possible bias of interpretation authors should compare results with all the participants.

We chose these two non-English speaking countries because they had the largest sample sizes. It is obvious to us that anyone using the kiosk in the first place is more than likely could read and understand English.

The information under the sub-section “Utility and accessibility of the website”, is not results! It could be information for the discussion section!

Note that we have removed all detailed information about the kiosk. We have moved the “Utility and accessibility of the website” to a supplemental file (Supplemental File 2).

In the discussion authors stated that “To date very few survey studies have attempted to assess the attitude of the public to microbes on a global scale.” and “The current study provides solid evidence that the global public do not fully understand the essential and beneficial role of microbes in health.”

Do authors believe that their study is a global scale? And provides evidence for global public? Do the visitors of museums represent the global public? This is an important bias of this study, and it should be discussed as an important limitation.

We address this below. In the context of the same issue raised by R2. The criticism that this is not a global survey but rather a survey of museum goers is well taken and we have refocused our take on who we have surveyed and what we call it..

Our major change to the text in response is the addition of a Limitations section which we hope clarifies some of the issues raised by both reviewers.

**Reviewer #2:** The authors do not seem to have a clear focus on what it is they want to report. The abstract (which is really a synopsis rather than an abstract) suggests they want to report on kiosk use, but the pros/cons/limitations of kiosk use are not discussed. The focus of discussion is on knowledge of microbes. They need to get their focus clearer.

This is a good point and one also mentioned by R1. To alleviate this problem we have focused the paper on results from the surveys and removed all of the information about kiosks to a supplemental file (Suppl File 3). We hope the focus is now clearer also from the introduction where we state that we focus entirely on answers to survey questions.

I had a major difficulty in reviewing in that the figures were so unclear as to be unreadable.

We have corrected and made clearer the figures. We don’t know why they were reproduced so inefficiently and unclearly.

The layout of the paper also made it difficult to read.

We have substantially reorganized the paper to reflect the comments of the reviewers

There needs to be discussion on the limitations of the methods.

We have added a “Limitations” section to the paper. This is indeed an important and essential part of this paper and we regret not having it in the first version of the paper.

See attached document for more commentary.

**Review of kiosk-based survey of microbe knowledge**

1. Calling the study a global survey seems a bit extravagant. It was a kiosk survey in a museum that has global visitors – but obviously those visitors are not typical of the countries that they come from, being wealthy enough to visit the USA and educated enough to want to visit a museum and ‘play’ with a kiosk in that museum. In the discussion again the kiosk users are referred to as “global public’s attitudes” – which is not accurate. The respondents are mostly tourists visiting New York are in no way representative of global knowledge or attitudes – they are probably not even representative of Americans from mid-western states who have not been to New York.

On reinspection we agree completely with the reviewer the the global statements we have made are probably not warranted. We agree that we do not have a true global sample here, but rather a sample of museum goers who on the average have a more complex and better educated view of scientific issues. However, this does mean that we are examining the “higher end” of the general public with respect to science issues (as we document with references about museum goers).

We have therefore softened the global aspect of the paper and completely understand the reviewer’s annoyance with our claim. But we do think it is important to report the differences amongst people from different countries no matter the socio-economic status of the respondents; especially when they do not differ drastically from country to country. We have changed this use of “global” sample to museum goers or entire sample.

2. Focus of the paper: In the abstract it says “This communication reports on the utility of kiosk survey methods in assessing public attitudes to microbes and microbe related issues” – implying that this is a main focus. Yet there as far as I can see there is no discussion about the use of kiosks versus (eg) doing a global internet survey as an alternative. There is no discussion about responses versus respondents (how many people tried the kiosk multiple times?), about whether the ‘episode of kiosk use’ was the same individual all the way through or whether for example (quite likely) groups of individuals were answering. What does the literature say about the uptake of opportunistic kiosk use?

We have drastically changed the abstract to reflect our focus on survey answers and not on kiosk efficacy. We agree completely that we sat the fence on whether the paper was about kiosks or peoples’ attitudes and knowledge and have shifted the focus of the paper to the more interesting aspect of knowledge and attitudes.

3. The abstract is not really an abstract but a synopsis – “This communication reports on …..” “….. we assess the relevance “. I want the abstract to tell me what you did, what the results are and what the implications are. This abstract does not do that.

We have revised the abstract to reflect WHAT WE DID! This is a great suggestion.

4. Age categories (page 16) can you clarify in which group people with ages 25 and 45 go? I presume it is 13 and less than 25, 25 and less than 45, 45 and less than 65?

I understand the problem. There are five age categories

<13

13 to 25

25 to 45

45 to 65

>65

These seemed like reasonable age demarcations from previous surveys accomplished at the AMNH. The visitors themselves submitted the age category they were in. So it is difficult to say which category 25, 45 and 65 year olds are in.

5. You said in the text that you used a mix of trade names and generic drug names in your questions. You referred to this in results saying (page 18) “although generic and specific names also were provided and **when possible**, we included generic names for these compounds that might be more familiar to the foreign visitors.” To find out how the questions were actually asked I had to delve down into the Figures/Tables.

(This may be PLOS ‘fault’ if they make you put the tables separate at the end of the article. Trying to read this onscreen it would have been MUCH better if it could have been laid out more like the finished journal article with the tables embedded in the text – **note to editor**). How the questions were asked is key to the paper.

This is a formatting problem that can easily be taken care of when then paper is typeset. Making sure that the survey questions are prominent and placed strategically is a good point of the reviewer. We want the questions to be in a table (Table 2) and set off from the text. In places we include the actual question and potential answers in the text.

This is important. On reading your text – before ‘finding’ Table 1 with the actual questions – I was thinking that I had never heard of the drug names Tynelol or Benadryl - on reading the questions as asked in Table 1 I was re-assured to some extent, even though this may still have ‘disadvantaged’ some countries. For example, I Googled antihistamine to find out what was available in India and found that antihistamines include: Brompheniramine (Dimetane), Cetirizine (Zyrtec), Chlorpheniramine (Chlor-Trimeton), Clemastine (Tavist), Diphenhydramine (Benadryl), Fexofenadine (Allegra), Loratadine (Alavert, Claritin). So it does include your one example (Benadryl) but in the UK (eg) the most familiar brand will be

Piriteze. I would expect to find this discussed in a limitations section of the discussion – but in fact

6. There is no LIMITATIONS section in your discussion.

We have added a limitations section, discussing many of the issues raised by the reviewers.

A. Skew caused by use of museum goers
B. Use of English language
C. Discussion of brand name limitations; This is indeed a problem we anticipated and tried to accommodate in our analyses. Basically if there is a brand name problem we would see it in the answers. But the general consistency of answers across countries indicates the respondents

.

7. The figures – at least in the pdf that I was asked to review – are of poor quality. It was also very difficult to have the (overlong) legends for the figures separated from the actual figures. So to try to understand a particular Figure I had to keep scrolling back and forth from the actual figure to the legend. The legends are almost mini-results sections rather than just legends. Many of the figures have lettering that I cannot read – the quality of images is too poor – maybe this is a result of the way the document has been saved and sent to reviewers – but without this I am unable to complete the review. I had no idea what Figure 2 was about until I was able to scroll back to the legend – it certainly is not what I was expecting to find at this point – I want to know how the questions were asked, not how you are now making your data available. Figure 3 blots out Tasmania and New Zealand.

Improved figures throughout the manuscript.

8. Hand sanitizers vs hand washing. One of the questions (Table 1) is about hand sanitizer but there appears nothing about hand washing, which seems very odd. Given that most people wash their hands with soap and water (at least in the UK) and use hand sanitizer only when available and hand washing is not possible – this seems a rather odd focus and not put into the perspective of a question about hand washing.

We focused in this paper on people’s perception and attitudes to microbes, and leave the subject of behaviors like hand washing and probiotics to future work.

9. Referencing (page 10) It does not look to me that Kartre is an appropriate reference for “….have been used in health education projects (Oppenheimer et al., 2011; Katre, 2009; Teolis, 2010; Samal et al., 2018),

Reference removed

**Minor typos/grammar issues noticed while reviewing**

10. The references are a mess in terms of their inconsistent and inappropriate non-use of capitals on journal names.

We note this with respect to the editor. Upon acceptance we will standardize the references for the final version.

11. On page 18 a whole load of abbreviations are used without prior introduction. I assume that these are geographical and are given in full on the Figure – but at that point I had not been introduced to what they meant (EU, LA, NA, AF, AS, IND, PAC). (And as a ‘Brit’ I recognise EU as (political) European Union but I guess here (probably) the authors are using it as (geographical) Europe.

The abbreviations are now clearly explained in the text of the paper and we included a sentence as to where the abbreviations can be found.

12. Sentence page 14: There are two basic ways to assess the frequency of correct and incorrect responses to objective questions. can be assessed (remove).

corrected

13. Page 19. In Survey 1, few of respondents used any – few respondents.

corrected
