# Supplementary material for: A natural history museum visitor survey of perception, attitude and knowledge (PAK) of microbes and antibiotics
Source: PLoS One. 2021 Sep 22;16(9):e0257085. doi: 10.1371/journal.pone.0257085 (PMC8457478; doi:10.1371/journal.pone.0257085)
Supplement: Attachment — Submitted filename: Response_reviewers.docx [file pone.0257085.s006.docx]

Response to reviewers

PONE-D-21-01340R1
A Natural History Museum Visitor Survey of Perception, Attitude and Knowledge (PAK) of Microbes and Antibiotics

There were two points you wanted us to address and these are in yellow below. Our response to the two points are in blue. You will see that we complied entirely with your requests.

In the previous round of review two reviewers noted that the following fragment was not supported by references "We point out that the results reported here are for museum goers, a category which is predominately better informed than others.". You have indicated in your response-to-reviewers that these references are provided in your manuscript. Whilst I understand that these are references are provided, this is some 6 paragraphs after the noted fragment. I therefore would be grateful if you could provide appropriate references immediately following the statement "We point out that the results reported here are for museum goers, a category which is predominately better informed than others."

I have added the museum-goer references in the proper place and shifted the reference numbers accordingly.

B) Your response to Reviewer 3 indicates that no formal analysis of the reliability/validity of the questions has been performed. Since this is an important part of any study using a questionnaire, please note the absence of these analyses in the limitations subsection of your Discussion

Inserted this statement in “Limitations” section:

Any study using a survey is based on the reliability and validity of the questions. While no formal analysis of these survey parameters were accomplished we suggest that our pre survey exploration addressed some of the validity concerns that surveys face. We also suggest that the consistency of similar answers from the survey done in 2017 with those from 2018 indicate a degree of reliability of those survey questions. Finally, we point out that several of the questions in our survey have been posed before in other surveys on antibiotics and microbial issues, which also suggesting a degree of validity of this study.

Thanks for moving this manuscript along. We feel it is now in very good shape after the two rounds of reviews and subsequent revisions. We fund the review process very helpful and feel strongly that it makes the paper much better and clearer. If you have any other requests about this manuscript please contact me.
